# Supplementary figures and images for: Absolute Proteome Quantification in the Gas-Fermenting Acetogen Clostridium autoethanogenum
Source: mSystems. 2022 Apr 6;7(2):e00026-22. doi: 10.1128/msystems.00026-22 (PMC9040625; doi:10.1128/msystems.00026-22)

**A**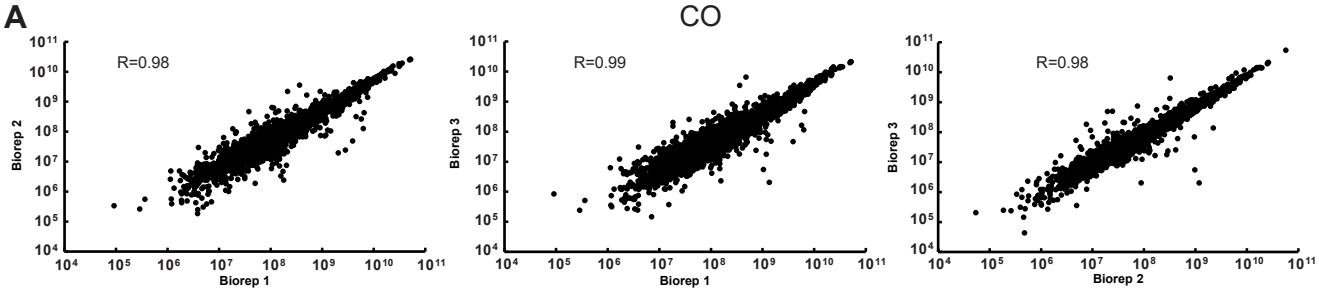**B**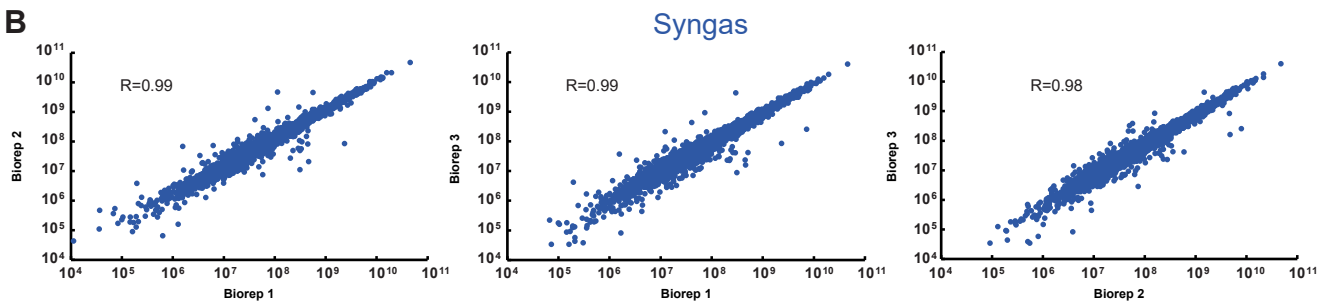**C**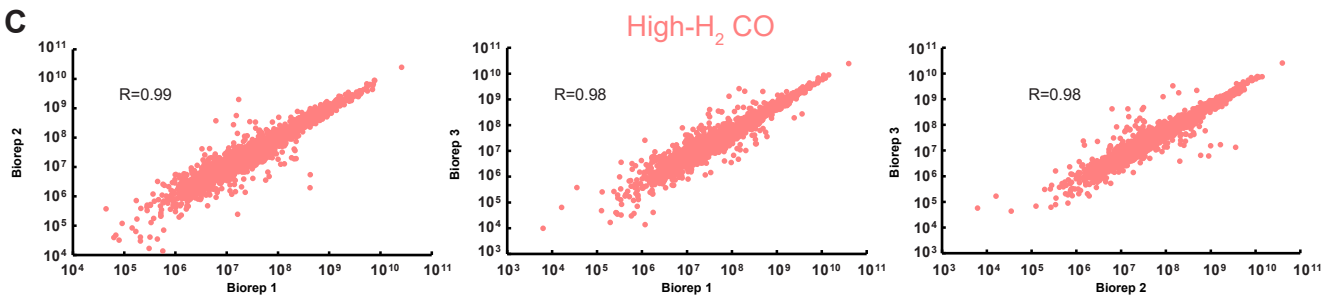

Supplement: FIG S1 [file msystems.00026-22-sf001.pdf]

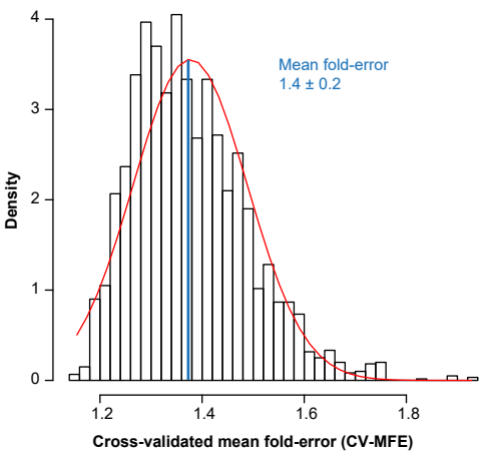

Supplement: FIG S2 [file msystems.00026-22-sf002.pdf]

Transcript abundance from  
Valgepea *et al.* 2017 (RPKM)

$R=0.65$   
#1033

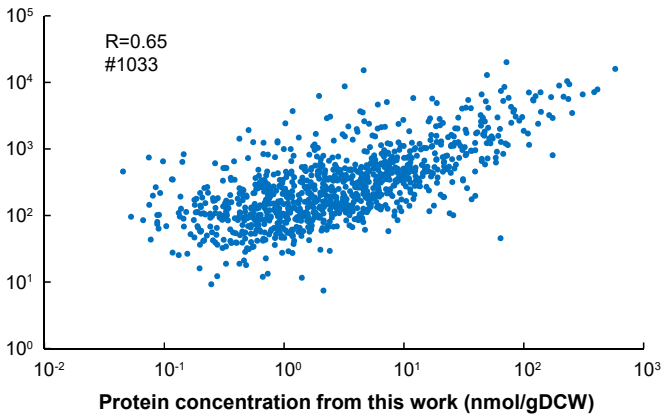

Supplement: FIG S3 [file msystems.00026-22-sf003.pdf]
