# Supplementary material for: Absolute Proteome Quantification in the Gas-Fermenting Acetogen Clostridium autoethanogenum
Source: mSystems. 2022 Apr 6;7(2):e00026-22. doi: 10.1128/msystems.00026-22 (PMC9040625; doi:10.1128/msystems.00026-22)
Supplement: FIG S4 [file msystems.00026-22-sf004.pdf]

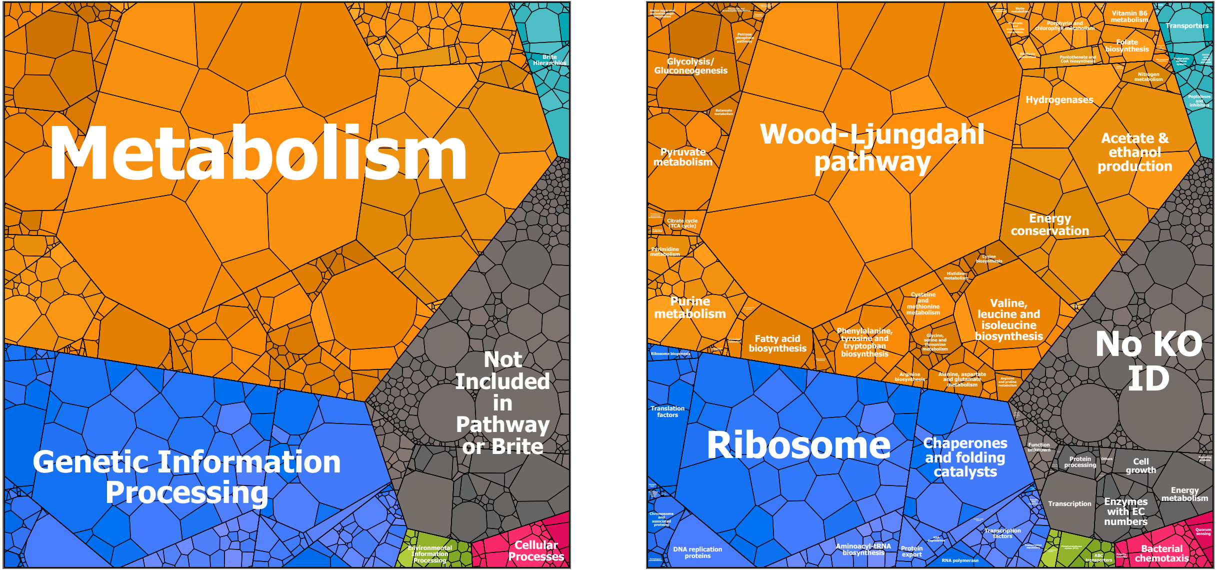

Metabolism
Brite Hierarchies
Not Included in Pathway or Brite
Genetic Information Processing
Environmental Information Processing
Cellular Processes
Glycolysis/ Gluconeogenesis
Pentose phosphate pathway
Butanoate metabolism
Pyruvate metabolism
Citrate cycle (TCA cycle)
Pyrimidine metabolism
Purine metabolism
Fatty acid biosynthesis
Wood-Ljungdahl pathway
Biotin metabolism
Porphyrin and chlorophyll metabolism
Vitamin B6 metabolism
Transporters
Folate biosynthesis
Pantothenate and CoA biosynthesis
Nitrogen metabolism
Peptidases and inhibitors
Hydrogenases
Acetate & ethanol production
Energy conservation
Lysine biosynthesis
Histidine metabolism
Cysteine and methionine metabolism
Phenylalanine, tyrosine and tryptophan biosynthesis
Glycine, serine and threonine metabolism
Valine, leucine and isoleucine biosynthesis
Arginine biosynthesis
Alanine, aspartate and glutamate metabolism
Arginine and proline metabolism
No KO ID
Ribosome biogenesis
Translation factors
Ribosome
Chaperones and folding catalysts
Function unknown
Protein processing
Others
Cell growth
Transcription
Enzymes with EC numbers
Energy metabolism
Chromosome and associated proteins
DNA replication proteins
Aminoacyl-tRNA biosynthesis
Protein export
RNA polymerase
Transcription factors
ABC transporters
Quorum sensing
Bacterial chemotaxis
